# VIRTual autOPSY—applying CT and MRI for modern forensic death investigations

**DOI:** 10.3389/fradi.2025.1557636

**Published:** 2025-05-12

**Authors:** Dominic Gascho

**Affiliations:** Institute of Forensic Medicine, University of Zurich, Zurich, Switzerland

**Keywords:** virtual autopsy (VIRTOPSY), postmortem computed tomography (PMCT), postmortem computed tomography angiography (PMCTA), postmortem magnetic resonance (PMMR) imaging, postmortem magnetic resonance spectroscopy, forensic imaging, postmortem imaging

## Abstract

Virtual autopsy, an advanced forensic technique, utilizes cutting-edge imaging technologies such as computed tomography (CT) and magnetic resonance imaging (MRI) to investigate the cause and manner of death without the need for physical dissection. By creating detailed, three-dimensional data of the entire body or specific areas of interest, these post-mortem imaging modalities provide a comprehensive, non-invasive approach to examining decedents. This article explores the historical development of virtual autopsy, its current applications in forensic medicine, and its promising future. It highlights the crucial roles of CT and MRI in forensic death investigations, while also addressing the challenges and limitations associated with these imaging techniques in post-mortem examinations.

## Introduction

A virtual autopsy is a modern, non-invasive forensic method for investigating causes of death using advanced imaging techniques, such as computed tomography (CT) and magnetic resonance imaging (MRI) (1–3). By capturing detailed three-dimensional representations of the entire body or specific regions, a virtual autopsy enables thorough documentation for subsequent reconstructions, measurements, and analyses. Unlike traditional autopsies, which involve physically examining and dissecting the body, a virtual autopsy allows investigators to visualize the internal structures and injuries of the body without making any incisions. This article explores the historical development of virtual autopsy, examines the current applications of post-mortem imaging in forensic medicine, and discusses the potential for future advancements. It highlights the roles of CT and MRI in forensic death investigations and addresses the challenges and limitations associated with these imaging technologies in their post-mortem use.

## Historical development: forensic radiography and modern post-mortem imaging in forensic medicine

**Figure 1:**
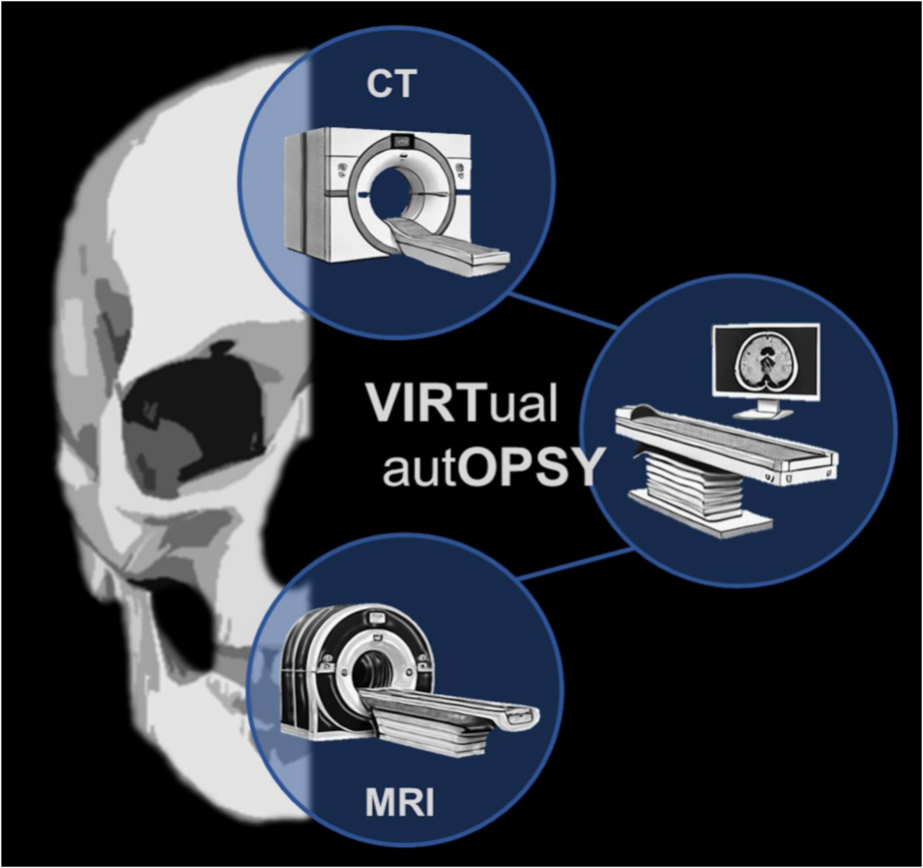
The term Virtopsy® (virtual autopsy) refers to the application of imaging technologies, such as CT and MRI, in forensic medicine to advance forensic death investigations. These post-mortem imaging techniques enable three-dimensional documentation of the entire body or specific regions, supporting accurate reconstructions and precise measurements.

Forensic radiography has been a cornerstone of investigative practices since the early 20th century. Remarkably, its legal application began just weeks after the discovery of x-rays, when a radiograph was presented as evidence in court (4). On December 24, 1895, in Montreal, Canada, Tolson Cunning was shot in the leg. Initial attempts to locate and retrieve the projectile were unsuccessful. However, a radiograph taken a few weeks later precisely identified the projectile's location, allowing surgeons to remove it. This radiograph was later introduced at the trial of the shooter, who was convicted and sentenced to prison. In the realm of post-mortem forensic imaging, radiographs have since been widely used to locate foreign objects, classify bone fractures, and identify remains through dental comparisons and the reconciliation of ante-mortem and post-mortem imaging (5). Like radiography, computed tomography (CT) imaging was first applied in gunshot cases shortly after its development. In 1977, a study was published detailing the assessment of gunshot wounds to the skull and their long-term consequences (6). During the 1990s, individual publications explored post-mortem applications of CT for detecting injuries and identifying deceased individuals (7–9). In 1994, Donchin et al. (7) proposed the use of post-mortem CT in trauma cases, stating, “*A possible way to circumvent the continuing decline in the number of autopsies is to perform computed tomography after death*”. Despite this early proposal, it took nearly a decade for the concept to gain momentum. A Swiss research team of forensic pathologists and radiologists advanced the field, introducing the term Virtopsy®—a blend of “virtual” and “autopsy”. This pioneering concept represents a significant innovation in forensic medicine, leveraging advanced imaging techniques such as CT and MRI to enhance or potentially replace traditional autopsies (Figure 1). This initiative was accompanied by several pioneering publications showcasing the application of CT and magnetic resonance imaging (MRI) in small-scale forensic cases (10–12). Since then, post-mortem three-dimensional imaging, particularly CT, has become an increasingly vital tool in forensic medicine.

## The role of post-mortem CT in forensic medicine: complement or replacement for conventional autopsy?

The question of whether post-mortem imaging can replace or merely complement conventional autopsy remains unresolved and continues to be scientifically debated. While post-mortem CT is increasingly performed by institutes as part of routine case work (13), its role as a replacement for conventional autopsy, or as a screening tool for minimally invasive autopsy, is still under scrutiny. This approach has both proponents and critics (14–19). Variations in outcomes may be attributed to differences in the capabilities of CT scanners, as well as the expertise of both the operator and the person interpreting the images. As with autopsy, post-mortem imaging relies heavily on visual diagnostics, making training and experience critical factors in its effectiveness (20). The diagnostic value of CT examinations also varies significantly due to the wide range of pathologies and injuries encountered in forensic medicine. This variability makes it challenging to make definitive statements regarding the overall use of post-mortem CT in forensic investigations. Additionally, comparing CT to autopsy is complex, as autopsies often include additional examinations such as toxicology and histology, which could be conducted alongside CT imaging through the collection of fluids or tissue samples (21–23). Moreover, even when a CT scan provides conclusive findings, it is generally insufficient for homicide investigations without an autopsy. In such cases, the autopsy remains a crucial component of a comprehensive forensic death investigation and continues to be regarded as the gold standard in forensic medicine (24). Since homicide cases are typically brought to court, a thorough death investigation, including autopsy, is essential.

While the conventional autopsy remains the gold standard in forensic death investigation, a complementary post-mortem CT scan is increasingly valued, particularly in trauma cases, as CT excels in detecting skeletal injuries (24). Additionally, CT is utilized even when no visible external injuries are present and a natural cause of death is suspected. In such cases, it is crucial to distinguish between natural causes of death and post-mortem “normal findings”—those resulting from autolysis and progressive decomposition (25). This distinction underscores the importance of specialized training and experience. There are also country-specific differences in which deaths are subject to further investigation by a forensic pathologist after an external examination, leading to significant institutional variation in the number of natural deaths that are investigated. Furthermore, access to CT scanners can influence which cases are imaged. Some institutes have their own CT scanners, while others rely on nearby hospitals, which necessitates additional coordination and makes imaging more selective, often focusing on trauma cases (13).

Although a post-mortem CT examination incurs financial costs, the data it provides offers significant forensic value for both the autopsy process and legal proceedings. Some examples of this value include:

▪ Infectious diseases detection: A non-invasive CT scan can help alert the forensic pathologist to specific infectious diseases (26).
▪ Radiological identification: CT data can be used to identify a deceased individual, particularly when DNA comparison is not possible due to the absence of relatives (27).
▪ Detection of air or gas inclusions: CT can visualize relevant findings such as air embolism in stab wounds, aiding in diagnosis (28).
▪ Localization of metallic foreign bodies: CT helps locate metallic foreign objects, such as projectiles or fragments, facilitating their recovery during the autopsy (29).
▪ Autopsy planning: CT reconstructions can assist in planning the autopsy, particularly for examining the wound channel in gunshot injuries (30).
▪ Fracture visualization: Volume rendering of CT data can present fractures and injuries in a format that is easily understood by non-medical professionals, such as prosecutors or in court (31).
▪ 3D printing for court presentation: CT data can be used to create 3D prints, allowing the presentation of physical representations of findings in court without the need to remove and macerate bone parts from the deceased (32).

Post-mortem CT has established itself as a valuable, non-invasive tool in forensic medicine (13). Its ability to examine the deceased without invasive procedures is particularly welcomed by some religious groups (1). However, despite its advantages, post-mortem CT has certain limitations. One significant drawback of virtual autopsy using radiologic techniques is that, in general, it lacks the sensory information provided by traditional autopsy. Unlike a physical examination, CT cannot offer visual color information (sight), tactile sensations (touch), or olfactory input (smell), all of which are crucial for the forensic pathologist. Moreover, metallic foreign bodies and medical implants made of metallic components can cause artifacts on CT images, complicating the assessment of surrounding soft tissues (33). Additionally, the contrast between soft tissues in CT images is relatively low due to the minimal physical differences in their densities, which can make it challenging to differentiate between certain tissue types and pathologies clearly.

## Post-mortem CT angiography: techniques, contrast agents, and diagnostic applications in forensic imaging

To optimize vascular contrast and to detect vascular lesions, blood vessels can be filled with a contrast agent mixture during a post-mortem CT angiography (CTA) (34). The literature describes various approaches regarding vascular access, filling technique, and the composition of the contrast agent mixture. For example, to achieve contrast enhancement from the level of the aortic bifurcation up to the cerebral vessels, access via the femoral artery is recommended (35). In contrast, for targeted coronary angiography, a selective approach via the subclavian artery is considered more appropriate (36). Access can be established by making a small incision and carefully exposing the vessel. After incising the vessel, a cannula can be inserted and secured using a vascular tourniquet. This procedure is performed separately for the arterial and venous vessels. Typically, the process begins with the arterial phase, during which the respective vessel is filled with contrast agent, followed by a CT scan. The same steps are then repeated for the venous phase (37). Filling can be carried out in various ways, by hand, with a standard clinical pump injector, with a roller pump system or with a conventional immersion pump (38, 39). The contrast medium mixture usually consists of an iodine-based CT contrast medium (for cost reasons, expired contrast medium is often used), which is mixed with a carrier substance such as polyethylene glycol (PEG) or Ringer's acetate solution, whereby the approximate mixing ratio of contrast medium to carrier substance is 1:20 (40). Each carrier substance has its own advantages and disadvantages, for instance, Ringer's acetate solution is more likely to cause extravasation, while PEG causes more severe changes to the organs and alters their haptic properties and color intensity, which can be relevant for subsequent autopsies (40). However, no adverse effects have been observed in later histological examinations (41). Post-mortem CTA is particularly suitable for the visualization of classic natural causes of death, such as an aortic dissection (Figure 3), and is valuable for detecting even the smallest vascular injuries by identifying the leakage of the contrast mixture (42, 43). However, when interpreting the resulting CT images, post-mortem changes must be taken into account. For example, individual blood clots in the vessels may be surrounded by the contrast agent mixture, which ultimately must not be misinterpreted for the radiological diagnosis (35). Nevertheless, in certain diagnostic scenarios, such as the precise localisation of a small vascular tear, post-mortem CTA can offer considerable added value and represent a powerful tool for virtual autopsy procedures (36).

## Post-mortem CT around the world: a global survey

**Figure 2:**
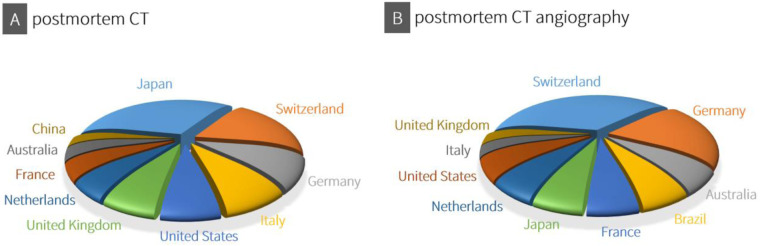
Using consistent data from both Scopus® (Elsevier®) and Web of science™ (Clarivate™) for the period from 2019 to 2024, an overview was compiled of the top 10 countries with scientific publications on CT (A: search terms “postmortem CT” and “PMCT”) and CTA (B: search terms “postmortem CT angiography” and “PMCTA”).

Although the use of post-mortem CT and CTA for forensic death investigations varies between countries due to regional practices, resources and legal frameworks, an overview of the top 10 countries with scientific publications on CT and CTA shows a global distribution of these post-mortem imaging techniques (Figure 2).

**Figure 3:**
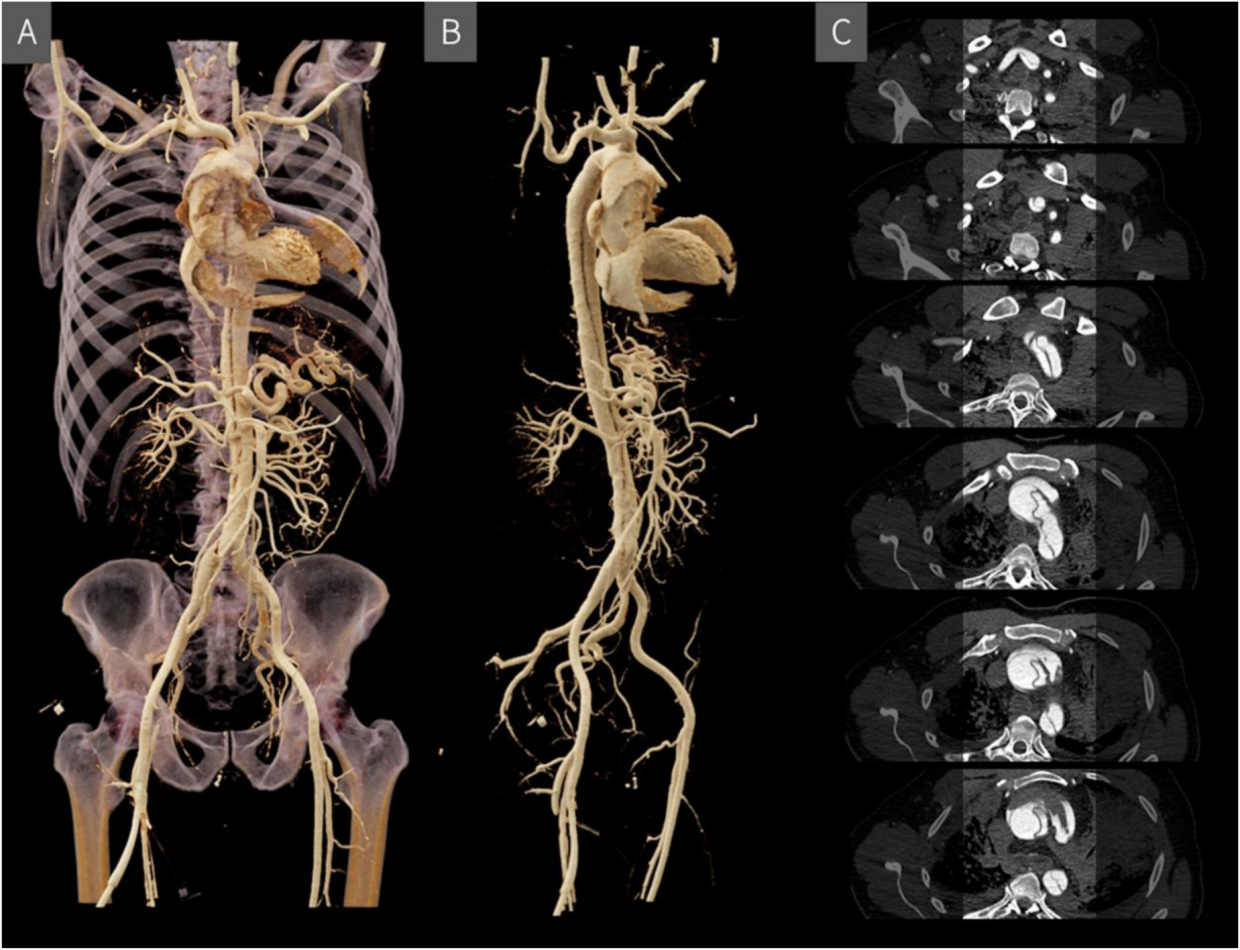
Post-mortem CT angiography visualized in 3D using cinematic rendering **(A** and **B)** and in individual cross-sectional slices **(C)** the rupture site is located above the valvular plane, extending ventrally with hemorrhage into the pericardial and mediastinal spaces. A Stanford type A aortic dissection originates at the level of the valvular plane/aortic root and continues supra-aortically, involving the brachiocephalic trunk and extending into the right subclavian artery, with a short segment also affecting the left subclavian artery.

In Japan, the declining rate of traditional autopsies, combined with the widespread availability of CT (and MRI) scanners, has led to the adoption of post-mortem imaging, also known as “autopsy imaging” (Ai) (44). Despite challenges such as the lack of radiologists trained in post-mortem imaging, the establishment of remote interpretation networks and government support highlight the growing importance of post-mortem imaging in modern forensic medicine in Japan (44). As noted in the historical introduction to this article, Switzerland is the birthplace of the Virtopsy®, and its university-affiliated institutes continue to conduct research in forensic post-mortem imaging while also defining medico-legal indications for its application (45, 46). Likewise, in Germany, post-mortem CT and CTA are becoming increasingly integrated into routine forensic practice (47). Apart from the United States, Australia, China, and, when it comes to CTA, also Brazil, the remaining countries among the illustrated top 10 are European. However, this top 10 illustration should not obscure the fact that other countries may also adopt post-mortem CT imaging more extensively in the future. For instance, India, where radiography is still commonly used in forensic investigations, recognizes the potential of three-dimensional CT imaging (48). Similarly, several African countries are beginning to implement post-mortem imaging, although in places like South Africa, full-body x-ray scanners (Lodox® systems) are more commonly used, and CT imaging is not yet widely established (49). For the global expansion of post-mortem CT imaging, affordable CT systems and modern implementation models could play a crucial role in integrating this technology into death investigation processes (50). Additionally, the continuous advancement of CT technology enhances the sensitivity and specificity of post-mortem CT imaging in forensic medicine.

## Advancing post-mortem CT imaging: future developments and applications in forensic medicine

Post-mortem imaging is expected to play an increasingly crucial role in forensic medicine in the coming years. New advancements in CT technology, particularly the development of modern photon-counting detector CT, offer exciting possibilities for the future of post-mortem imaging. This technology promises to reduce image noise and allow for a slice thickness of just 0.2 mm (51). The resolution of post-mortem CT scans is likely to advance from macroscopic to mesoscopic levels, enabling more detailed visualizations of certain forensically relevant findings (52). Achieving this level of resolution across the entire body is currently impractical due to the enormous data sizes, and the visual diagnosis of individual layers may not be feasible at this stage. In addition to high resolution, the new photon-counting detectors offer promising advancements in material differentiation through spectral data. This data can, for example, be used to assess bone density (53, 54), a valuable factor when analyzing fractures caused by blunt force trauma. Bone density can vary significantly between individuals, and understanding this variability can help accurately assess the force required to cause specific fractures. Furthermore, bone density information plays a crucial role in the development of virtual impact force calculations using finite element analysis (FEA). FEA is a computer-aided method used to simulate and analyze the physical behavior of structures under various conditions (55). In the context of trauma, FEA is especially useful for understanding the mechanics of injuries, predicting outcomes, and improving protective measures. Subject-specific finite element models could become an invaluable tool for forensic medicine, enhancing the precision and depth of trauma assessments (56). In addition to high-end clinical radiology CT scanners, more affordable alternatives are being explored to enable institutions with limited budgets to access this valuable imaging technology, though with certain limitations. One such alternative is the use of mobile CT scanners, originally designed for radiotherapy and intraoperative procedures, which have recently been proposed for post-mortem imaging (57). Regardless of cost considerations, it is important to test various systems for their suitability in forensic medicine, as post-mortem imaging has specific requirements. For example, a larger gantry diameter is desired for CT scanning of charred bodies, as the deceased often remains rigid in the post-mortem pugilistic posture—a “boxer-like” position with bent elbows and knees, and clenched fists. This phenomenon results from the shrinkage of body tissues and muscles due to dehydration caused by the heat.

## Virtual autopsy in practice: addressing training and expertise challenges in post-mortem imaging

Collaboration between radiologists and forensic pathologists is crucial for accurate and comprehensive analysis in post-mortem imaging, particularly in complex cases (24). Teleradiology could be a viable solution for handling more complex cases when the direct employment of additional radiologists in forensic services is not financially feasible. While radiologists are highly skilled in CT diagnostics, post-mortem imaging differs significantly from clinical patient scans (58). Forensic pathologists, on the other hand, are experts in death investigations but typically lack specialized training in CT imaging. Therefore, specialized training in post-mortem imaging is essential for both forensic pathologists and radiologists to ensure accurate diagnostic outcomes (59–61). Currently, there are only a limited number of training courses available for the radiological interpretation of post-mortem CT data, though studies have been published on adapting scan and reconstruction parameters for post-mortem CT examinations (33, 37, 52). A recent article outlines various models for managing and operating post-mortem imaging services, including those led by pathologists, radiologists, or a combination of both (50). It also explores the use of stationary, relocatable, and mobile CT scanning units, raising an important question: who should perform the CT scans? Ideally, radiologic technologists with additional forensic training are preferred, but due to staffing constraints, CT scans are often carried out by autopsy technicians (13, 62). With regard to post-mortem MRI examinations, radiologic technologists and radiologists remain indispensable for conducting the procedures and performing diagnoses.

## The evolving role of MRI in post-mortem imaging: advantages, challenges, and future directions

**Figure 4:**
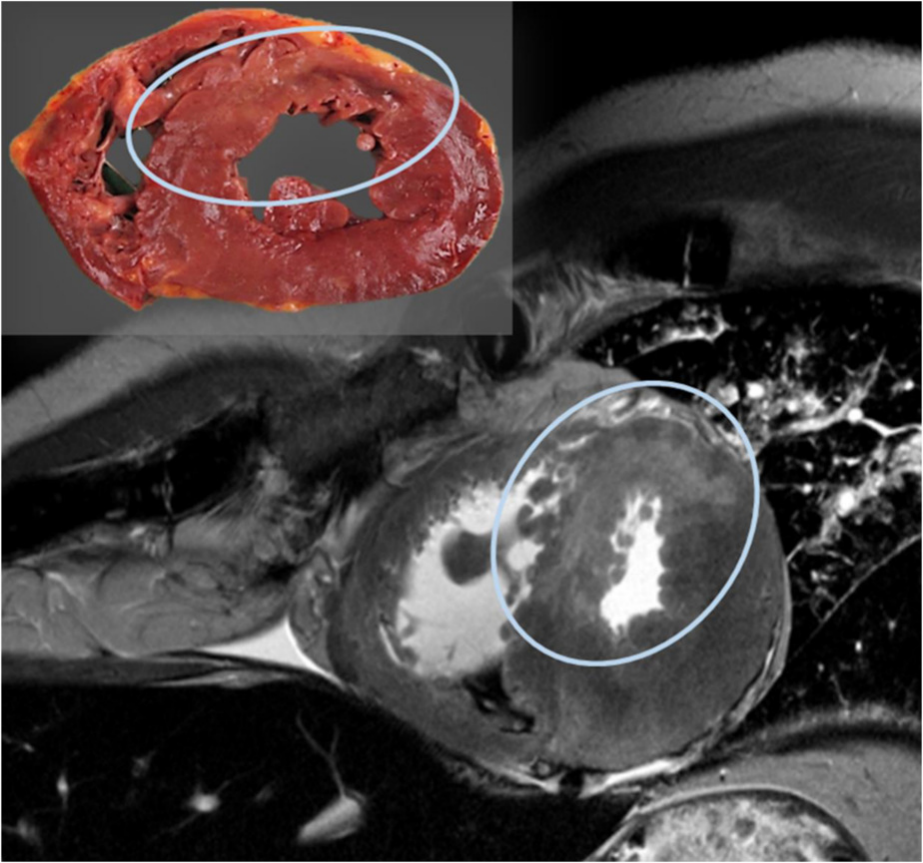
Post-mortem MRI examination of the heart shows a hyper-intense (bright) region in the septum and parts of the anterior wall of the left ventricle on the T2-weighted image, indicating myocardial edema; the corresponding autopsy slice is located in the upper left.

For the visualization of soft tissue injuries and specific organ pathologies, MRI provides significant advantages over CT. The literature highlights its use, for example, in detecting bleeding in the neck muscle after strangulation in both living and deceased individuals, or identifying acute and even subacute myocardial infarction (Figure 4), which is often not detectable through autopsy (63–67).

However, post-mortem MRI remains relatively uncommon and is primarily employed in select cases, typically as a complement to CT. This is due, in part, to the fact that MRI examinations are time-consuming, the image contrast is complex to produce, and the operation of the scanner requires careful attention, especially due to potential complications with ferromagnetic foreign bodies exposed to the strong magnetic field (68). Furthermore, only a small number of institutes worldwide can afford both CT and MRI scanners, often prioritizing the use of the more readily accessible CT scanner (68). Compared to CT, adapting MRI from clinical radiology to post-mortem imaging also presents significantly greater challenges. The contrast in MRI is heavily influenced by body temperature and decomposition processes, which complicates image interpretation. While efforts have been made to account for these factors, more research is needed to understand and measure the impact of decomposition on MRI contrast (69–71). Despite these obstacles, post-mortem MRI is increasingly being utilized for pediatric examinations in both forensic medicine and pathology (72–75). This is likely due to the low soft tissue contrast in CT scans of infants and young children, making MRI the preferred choice. Additionally, there is a strong preference for avoiding autopsies in children wherever possible. Recommendations and guidelines for pediatric post-mortem MRI examinations are available in the literature (76, 77). However, the known challenges of post-mortem MRI related to body temperature and decomposition processes also apply to pediatric examinations. Despite these challenges, MRI offers high sensitivity and specificity for a wide range of post-mortem fetal diagnoses, especially when combined with other non-invasive techniques, and is particularly effective for detailed soft tissue assessments (78). Regarding the common perception that post-mortem MRI examinations are time-consuming, post-mortem imaging benefits from the clinical demands driving advancements in MRI technology. In clinical radiology, there has been an increasing demand for shorter examination times, and sensitive encoding techniques, which significantly reduced examination time from the 2000s onward (79). Today, further reductions in examination time are expected through the application of artificial intelligence (80).

## Ultra-high-field MRI scanners: emerging opportunities for forensic medicine

The development of ultra-high-field MRI scanners represents a remarkable step forward in imaging technology. In recent years, 7 Tesla (7T) MRI scanners have been approved for patient examinations (81). The diagnostic superiority of 7T over 3T images for brain imaging in post-mortem examinations has been demonstrated in individual cases (82, 83). For instance, 7T imaging has shown exceptional clarity in depicting microbleeds in the brain (83). While these microbleeds are also visible in 3T images, the more pronounced blurring in 3T images increases the risk of misidentification or oversight. In another case, 7T MRI provided a striking visualization of coup and contre-coup injuries resulting from a fatal fall down a flight of stairs (83). Coup and contre-coup injuries refer to brain trauma where the coup injury occurs directly at the site of impact, and the contre-coup injury occurs on the opposite side due to the brain striking the inner skull. Additionally, post-mortem examinations of three cases involving cerebral gunshot wounds highlighted the utility of 7T MRI in identifying injuries related to the extent of the temporary wound cavity (82). A temporary wound cavity in gunshot injuries refers to the transient, rapidly expanding cavity created by the kinetic energy transfer from a projectile, causing surrounding tissues to stretch violently beyond their elastic limits. The enhanced susceptibility effect at 7T enabled a more sensitive detection of smaller hemorrhages in the cerebral tissue surrounding the wound channel compared to 3T imaging. Although the high cost and logistical challenges of installing ultra-high-field MRI scanners make them unlikely additions to forensic medicine institutes, their selective use in hospital settings could be highly valuable in the future. For example, access to 7T MRI for specific cases, such as detecting subtle shearing injuries in suspected child abuse, could significantly enhance forensic investigations and provide critical insights in complex cases.

## Beyond the imaging: exploring the potential of post-mortem magnetic resonance spectroscopy (MRS)

In addition to its imaging capabilities, MRI units also offer the possibility of conducting analytical examinations through magnetic resonance spectroscopy (MRS). MRS is a non-invasive technique used to investigate metabolic changes in tissues, particularly in the brain. Unlike MRI, which provides detailed images of anatomical structures, MRS focuses on the biochemical environment within tissues, offering a spectrum that represents the concentrations of various metabolites at different chemical shifts. Recent studies have highlighted several potential applications of MRS for post-mortem examinations. For example, MRS can be used to detect fatal metabolic disorders, which are traditionally identified through vitreous humor samples analyzed in clinical biochemistry (84). Moreover, MRS can measure ethanol levels non-invasively in the brain, where the substance has a longer detection window compared to blood samples, and where it directly affects consciousness (85). Further forensic studies have demonstrated that MRS can detect metabolic changes in bone marrow lipids following freezing and thawing of severed sheep legs, potentially indicating exposure to very low temperatures (86). While these findings following freezing and thawing have not yet been confirmed in human post-mortem cases, they clearly showcase the promising capabilities of MRS. In addition, MRS can measure tissue temperature through a technique known as MRS thermometry. This method takes advantage of the temperature dependence of hydrogen bonds to determine cerebral temperature, allowing for non-invasive temperature measurement in the brain during MRI scans (87). Thus, post-mortem MRS offers exciting new possibilities for virtual autopsies, providing analytical insights without the need for invasive sample collection. Although its widespread use in routine forensic work is still a long way off, MRS has the potential to revolutionize post-mortem examinations. In addition to access to MRI technology, the successful implementation of MRS in forensic practice would require specialized expertise to accurately analyze the data. In the future, biomedical engineers and medical physicists will play an increasingly vital role in post-mortem imaging. While these professionals typically collaborate with radiologists to develop equipment and examination protocols tailored to the needs of clinical radiology, their involvement has been limited in forensic medicine. In forensic settings, there is a need for these experts to collaborate with forensic physicians to meet the unique demands of post-mortem investigations and to further develop imaging techniques tailored for forensic purposes.

## Summary: the current state and future potential of post-mortem imaging in forensic medicine

Currently, post-mortem imaging is primarily used to complement traditional autopsies. CT scans, in particular, are becoming increasingly popular in forensic medicine due to their ability to rapidly document the entire body in just a few seconds. MRI, particularly when combined with MRS, offers a broad range of potential applications but remains challenging to implement in forensic medicine due to its complexity. Additionally, the high cost of MRI equipment, which usually exceeds that of CT scanners, presents another barrier. Nevertheless, these imaging technologies will continue to evolve and are expected to solidify their role in forensic imaging. The future of post-mortem imaging in forensic death investigation is promising, driven by continuous technological advancements and innovative adaptations for post-mortem applications. The potential of these imaging techniques to reveal critical information regarding the manner and cause of death is far from being fully realized. It remains to be seen which of these emerging applications will transition from experimental or scientific use into routine forensic casework.
